# Baseline Participant Characteristics and Risk for Dropout from 10 Obesity Randomized Controlled Trials: A Pooled Analysis of Individual Level Data

**DOI:** 10.3389/fnut.2014.00025

**Published:** 2014-12-18

**Authors:** Kathryn Ann Kaiser, Olivia Affuso, Renee Desmond, David B. Allison

**Affiliations:** ^1^School of Public Health, University of Alabama at Birmingham, Birmingham, AL, USA; ^2^Nutrition Obesity Research Center, University of Alabama at Birmingham, Birmingham, AL, USA; ^3^Department of Epidemiology, School of Public Health, University of Alabama at Birmingham, Birmingham, AL, USA; ^4^Department of Preventive Medicine, School of Medicine, University of Alabama at Birmingham, Birmingham, AL, USA

**Keywords:** obesity, pooled analysis, randomized trials, dropout, participant characteristics

## Abstract

**Introduction:** Understanding participant demographic characteristics that inform the optimal design of obesity randomized controlled trials (RCTs) have been examined in few studies. The objective of this study was to investigate the association of individual participant characteristics and dropout rates (DORs) in obesity RCTs by pooling data from several publicly available datasets for analyses. We comprehensively characterize DORs and patterns in obesity RCTs at the individual study level, and describe how such rates and patterns vary as a function of individual level characteristics.

**Methods:** We obtained and analyzed nine publicly available, obesity RCT datasets that examined weight loss or weight gain prevention as a primary or secondary endpoint. Four risk factors for dropout were examined by Cox proportional hazards including sex, age, baseline BMI, and race/ethnicity. The individual study data were pooled in the final analyses with a random effect for study, and HR and 95% CIs were computed.

**Results:** Results of the multivariate analysis indicated that the risk of dropout was significantly higher for females compared to males (HR = 1.24, 95% CI = 1.05, 1.46). Hispanics and Non-Hispanic blacks had a significantly higher dropout rate compared to non-Hispanic whites (HR = 1.62, 95% CI = 1.37, 1.91; HR = 1.22, 95% CI = 1.11, 1.35, respectively). There was a significantly increased risk of dropout associated with advancing age (HR = 1.02, 95% CI = 1.01, 1.02) and increasing BMI (HR = 1.03, 95% CI = 1.03, 1.04).

**Conclusion/Significance:** As more studies may focus on special populations, researchers designing obesity RCTs may wish to oversample in certain demographic groups if attempting to match comparison groups based on generalized estimates of expected DORs, or otherwise adjust *a priori* power estimates. Understanding true reasons for dropout may require additional methods of data gathering not generally employed in obesity RCTs, e.g., time on treatment.

## Introduction

Dropout is a major problem in studies of weight loss interventions (1). Identification of predictors of dropout could be important to enhance recruitment in vulnerable groups, as well as to develop strategies to prevent dropout among those at high risk. Previous investigations in single studies have reported baseline factors that are associated with dropout including sex, age, marital status and race, e.g., Ref. (2), or the presence of baseline comorbidities such as Type 2 diabetes (3). Psychological predictors of dropout such as motivation and stages of change have also been investigated as factors, with little evidence of reliable predictive value across multiple studies for many of the variables proposed (4). The purpose of this investigation is to conduct a pooled meta-analysis to identify baseline factors that are related to study retention among a large cohort of subjects with racial/ethnic, age, sex, and body weight heterogeneity.

## Materials and Methods

### Study samples

For this investigation, individual level participant data from the selected studies were obtained from the Biologic Specimen and Data Repository Information Coordinating Center (BioLINCC), for the National Heart, Lung, and Blood Institute (5) and from the National Institute for Digestive and Diseases of the Kidney (NIDDK) Central Data Repository (https://www.niddkrepository.org). Searches were performed for studies meeting the inclusion criteria defined as: the interventions were dietary and/or physical activity interventions in free living people of any age, an outcome of interest was body weight, and basic demographic information such as age, gender, and race were available in most records. Ten were selected for inclusion (6–16). The investigation was approved for secondary data analysis by the Institutional Review Board at the University of Alabama at Birmingham.

### Data definitions and recoding

The raw, de-identified datasets obtained were standardized for consistency in coding prior to pooling. Variable codes were assigned as follows: sex (0 = male, 1 = female); race (0 = White, Non-minority or non-Black, 1 = Black, 2 = Asian, 3 = Hispanic, 4 = other); age (continuous coded in years); body mass index (BMI – continuous); dropout (0 = No, 1 = Yes for any time before the protocol was completed), follow-up time (months). Treatment groups were coded as nominal variables within each study. The individual study frequency data were examined and compared to study publications for accuracy (6–16).

### Statistical analysis

Descriptive statistics including means and standard deviations (SD) for continuous data and frequency counts for categorical predictors were generated by study as well as for the overall analyses. The Cox model was used to estimate hazard ratios (HR) for risk of dropout within each study. Proportionality assumptions were assessed within each study by including a predictor term in the model for the interaction of the predictors with time. If a time-dependent covariate was significant, this could indicate a violation of the proportionality assumption for that specific predictor. A Martingale residual analysis was used to examine whether the functional form of the linear predictors was appropriate or whether a quadratic term would improve model fit (17, 18).

For the combined analysis, the participant-level raw data were pooled from the multiple studies. Two types of pooled analyses were performed. The preliminary analyses combined the study-specific HR and standard errors using the SAS METAANAL macro (18) to produce the DerSimonian-Laird (19) estimator for random effects. Plots of the study specific estimates as well as the overall random effects model were visually inspected for heterogeneity among the studies for estimates of BMI, age, sex, and race/ethnicity. Due to the significant heterogeneity detected on the random effects models for univariate predictors, the final pooled model using a combined dataset was run with PHREG^®^ (SAS Ver. 9.2, Cary, NC, USA) using study as a random effect. Further multivariate Cox models were analyzed to include categorical terms for BMI (<35, 35–39.9, ≥40) and age (<25, 25–64.9, ≥65) while controlling for sex and race/ethnicity.

## Results

Table 1 summarizes the studies included in the meta-analysis including a brief description, the endpoint for determining censoring for dropout and the dropout proportion. The descriptive characteristics for the covariates included in this investigation are shown in Table 2. The results of the pooled analyses are in Table 3 (using age and BMI as continuous variables) and in Table 4 (using age and BMI as categorical variables).

**Table 1:** **Endpoint determination and reference used for included studies**.

Study title	Study description	Dropout determination	Dropout *n* (%)	Published missingness *n* (%)
Activity Counseling Trial (ACT) (11)	The ACT was a multicenter, randomized, controlled trial to evaluate the effects of two primary care based activity counseling regimens on physical activity and cardiorespiratory fitness after 24 months when compared with a standard.	Subjects who did not complete assessment at 2 years	81 (9.3)	75 (8.6) had no assessment at 24 months
Dietary Approaches to stop hypertension (DASH) (10)	The DASH trial was a multicenter, randomized feeding study that tested the effects of dietary patterns on blood pressure. The intervention phase was an 8-week period in which the subjects followed their assigned diets.	Subjects who did not complete the assessment at 8 weeks	17 (3.7)	13 (2.8) percentage who did not complete intervention phase
Dietary Intervention Study in Children (DISC) (7)	The DISC study was a controlled clinical trial to examine the efficacy and safety of long-term dietary intervention for reduction of LDL-C in pubescent children.	Subjects who did not attend the 3-year lipid assessment visit	43 (6.5)	39 (5.9) number with no outcome data at 3 years
Diabetes Prevention Program (DPP) (13)	A randomized trial was conducted to compare a lifestyle intervention or metformin on the development of diabetes.	The blinded treatment was terminated early so dropout was assessed by an algorithm provided by the investigator (personal communication to author Renee Desmond)	149 (4.1)	246 (7.6) percentage who had no follow-up within last 5 months
Lipids Research Clinics (LRC) Coronary Primary Prevention Trial (14)	The LRC CPPT was a multicenter, randomized, double-blind clinical trial on the efficacy of cholesterol lowering.	Subjects who did not complete the minimum follow-up of 7 years	214 (5.7)	Not reported
Multiple Risk Factor Intervention Trial (MRFIT) (12)	MRFIT was a randomized, primary prevention trial to test whether lowering elevated serum cholesterol and diastolic blood pressure and ceasing cigarette smoking would reduce coronary heart disease mortality.	Subjects who did not complete a follow-up period of at least 6 years	1003 (7.8)	Not reported
PREMIER (9)	The objective of the randomized trial was to determine the effect on blood pressure to two multi-component behavioral interventions.	Subjects who did not complete the primary outcome assessment at 6 months and did not complete the 18-month intervention	139 (17.2)	45 (5.5) did not complete 6 months follow-up although intervention lasted 18 months
Trials of Hypertension (TOHPI Lifestyle and II) (6)	Three lifestyle change groups were compared with unmasked non-intervention controls over 18 months to assess change in diastolic blood pressure. Phase II TOHP was a multicenter randomized controlled clinical trial with a 2 × 2 factorial design.	Subjects who did not complete an 18-month follow-up visit (Lifestyle TOHP I) or subjects who did not complete a 36-month follow-up visit (TOPH II)	92 (6.3) 243 (10.2)	37 (4.2) of active arms did not complete follow-up 273 (11.5) did not have weight at 36 months
Women’s Health Initiative (WHI) Dietary Modification Trial (15)	The WHI dietary modification trial was designed to examine the benefits and risk of a dietary pattern low in fat on various outcomes in postmenopausal women.	Subjects who were deceased or who stopped follow-up before 7 years	2242 (4.6)	4071 (8.3) withdrew, lost, or deceased

**Table 2:** **Characteristics of included studies**.

Factor	ACT	DASH	DISC	DPP	LRC	MRFIT	PREMIER	TOHP I	TOHP II	WHI
Gender										
Male	479 (54.8)	234 (51.0)	362 (54.6)	362 (54.6)	3,774 (100.0)	12,866 (100.0)	310 (38.3)	1,037 (70.7)	1,566 (65.7)	0 (0.0)
Female	395 (45.2)	225 (49.0)	301 (45.4)	301 (45.4)	0 (0.0)	0 (0.0)	500 (61.7)	429 (29.3)	816 (34.3)	48,835 (100.0)
Race/ethnicity										
Non-Hispanic White	597 (68.9)	156 (34.0)	574 (86.6)	2,117 (57.8)	3,601 (96.6)	11,559 (89.8)	531 (65.6)	1,182 (80.6)	1,888 (79.3)	39,762 (81.6)
Non-Hispanic Black	217 (25.0)	303 (66.0)	56 (8.5)	751 (20.5)	125 (3.4)	931 (7.3)	279 (34.4)	252 (17.2)	421 (17.7)	5,262 (10.8)
Asian	34 (3.9)	0 (0.0)	33 (5.0)	0 (0.0)	0 (0.0)	0 (0.0)	0 (0.0)	0 (0.0)	73 (3.1)	1,105 (2.3)
Hispanic	0 (0.0)	0 (0.0)	0 (0.0)	609 (16.6)	0 (0.0)	0 (0.0)	0 (0.0)	0 (0.0)	0 (0.0)	1,845 (3.8)
Other	19 (2.2)	0 (0.0)	0 (0.0)	188 (5.1)	0 (0.0)	376 (2.9)	0 (0.0)	32 (2.2)	0 (0.0)	762 (1.6)
BMI										
<18	4 (0.5)	0 (0.0)	459 (69.2)	0 (0.0)	6 (0.2)	21 (0.1)	0 (0.0)	5 (0.3)	0 (0.0)	86 (0.2)
18.5–24.9	210 (24.3)	114 (24.8)	202 (30.5)	0 (0.0)	1,215 (32.5)	2,649 (20.6)	39 (4.8)	354 (24.2)	24 (1.0)	12,551 (25.8)
25.0–29.9	303 (35.1)	179 (39.0)	2 (0.3)	1,199 (32.7)	2,215 (59.2)	7,125 (55.4)	242 (29.9)	694 (47.3)	1,014 (42.6)	17,425 (35.8)
≥30	346 (40.1)	166 (36.2)	0 (0.0)	2,466 (67.3)	305 (8.1)	3,071 (23.9)	529 (65.3)	413 (28.2)	1,344 (56.4)	18,591 (38.2)
*Obese class I	208 (59.2)	158 (95.2)	0 (0.0)	915 (37.1)	305 (100.0)	2659 (86.6)	241 (45.5)	372 (90.1)	1,048 (78.0)	11,216 (60.3)
*Obese class II	89 (23.6)	8 (4.8)	0 (0.0)	940 (38.1)	0 (0.0)	404 (13.2)	469 (32.0)	41 (9.9)	296 (22.0)	5,046 (27.1)
*Obese class III	49 (14.5)	0 (0.0)	0 (0.0)	611 (24.8)	0 (0.0)	8 (0.2)	119 (22.5)	0 (0.0)	0 (0.0)	2,329 (12.5)
Age										
<29	0 (0.0)	49 (10.7)	663 (100.0)	0 (0.0)	0 (0.0)	0 (0.0)	11 (1.4)	0 (0.0)	0 (0.0)	0 (0.0)
30–49	434 (49.7)	283 (61.6)	0 (0.0)	1,811 (49.4)	2,257 (60.3)	8,670 (67.4)	426 (52.6)	1,162 (79.3)	1,899 (79.7)	2 (0.0)
50–69	403 (46.1)	118 (25.7)	0 (0.0)	1,854 (50.6)	1,487 (39.7)	4,196 (32.6)	359 (44.3)	304 (20.7)	483 (20.3)	40,711 (83.4)
>70	37 (4.2)	9 (2.0)	0 (0.0)	0 (0.0)	0 (0.0)	0 (0.0)	14 (1.7)	0 (0.0)	0 (0.0)	8,122 (16.6)
BMI (mean, SD)	29.4 (6.0)	28.2 (3.8)	17.5 (2.4)	33.5 (95.7)	26.2 (2.6)	27.7 (3.5)	33.1 (5.7)	27.8 (3.7)	30.9 (3.1)	29.1 (5.7)
Age (mean, SD)	50.9 (10.0)	44.6 (10.7)	9.6 (0.7)	50.5 (9.3)	47.2 (6.5)	46.2 (6.0)	50.0 (9.1)	43.0 (6.5)	43.6 (6.2)	62.3 (6.9)

*n (%) within obese.

*ACT, Activity Counseling Trial (11); DASH, Dietary Approaches to Stop Hypertension (10); DISC, Dietary Intervention Study in Children (7); DPP, Diabetes Prevention Program (13); LRC, Lipids Research Clinics Coronary Primary Prevention Trial (14); MRFIT, Multiple Risk Factor Intervention Trial (12); PREMIER (9); TOHPI, Trials of Hypertension (Lifestyle I and II) (6) WHI, Women’s Health Initiative Dietary Modification Trial (15)*.

*All values *n* (%)*.

**Table 3:** **Results of pooled analysis of individual data for dropout risk by baseline characteristics (*n* = 75,764)**.

Factor	Parameter estimate	Standard error	*p*	HR (95% CI)
Gender
Male	–			1
Female	0.21435	0.08517	0.0118	1.24 (1.05, 1.46)
Race
Non-Hispanic White	–	–	–	1
Non-Hispanic Black	0.20127	0.04888	<0.0001	1.22 (1.11, 1.35)
Asian	0.21775	0.13263	0.1006	1.24 (0.96, 1.61)
Hispanic	0.48028	0.08424	<0.0001	1.62 (1.37, 1.91)
Other	0.24451	0.11038	0.0268	1.28 (1.03, 1.56)
Age (years)	0.01446	0.00237	<0.0001	1.02 (1.01, 1.02)
BMI (units)	0.03097	0.00303	<0.0001	1.03 (1.03, 1.04)

**Table 4:** **Results of pooled analysis of individual data for dropout risk by baseline characteristics using categorical variables for age and BMI (*n* = 75,764)**.

Factor	Parameter estimate	Standard error	*P*	HR (95% CI)
Gender
Male	–			1
Female	0.19961	0.08539	0.0194	1.22 (1.03, 1.44)
Race
Non-Hispanic White	–	–	–	1
Non-Hispanic Black	0.22306	0.04874	<0.0001	1.25 (1.14, 1.38)
Asian	0.16679	0.13237	0.2077	1.18 (0.91, 1.53)
Hispanic	0.50321	0.08426	<0.0001	1.65 (1.40, 1.95)
Other	0.24367	0.11039	0.0273	1.28 (1.03, 1.58)
Age (years)
<25 years	1.19314	0.6954	0.0862	3.30 (0.84, 12.86)
25–64 years	–	–	–	–
≥65 years	0.31667	0.04184	<0.0001	1.37 (1.26, 1.49)
BMI (units)
<35 Class I or less	–	–	–	–
35–40 Class II	0.33444	0.05248	<0.0001	1.39 (1.26, 1.55)
>40 Class III	0.52731	0.07047	<0.0001	1.69 (1.48, 1.95)

The results of univariate models including single factors in the model for each study are shown in Tables 5–8. The testing of residuals for age and BMI in each study did not show a significant departure from expected simulations. In the Dietary Intervention Study in Children (DISC) study, there was some indication of a poor fit for a linear model for age, although this study involved children aged 8–10 years old. The proportionality assumptions for the interaction terms of gender by time, race by time, BMI by time, and age by time were tested within each study. The results showed a statistically significant interaction for gender by time in the Dietary Approaches to Stop Hypertension (DASH) study and TOHP1 study, and race by time in the Diabetes Prevention Program (DPP) study. No other interaction terms were significant.

**Table 5:** **Summary of effect estimates for Body Mass Index (BMI) by study and estimates of effects in meta-analyses of dropout**.

Study	Parameter estimate	Standard error	Weight in fixed effects model	Weight in random effects model
ACT	0.07021	0.01676	0.02754	0.11135
LRC	−0.01250	0.02669	0.01086	0.05682
DASH	−0.04058	0.06338	0.00193	0.01196
DISC	0.16774	0.05975	0.00217	0.01339
DPP	−0.01343	0.00467	0.03471	0.12785
MRFIT	0.02471	0.01028	0.07321	0.18225
PREMIER	0.02035	0.01529	0.03309	0.12439
TOHP I	0.01138	0.03193	0.00759	0.04213
TOHP II	0.00347	0.02925	0.00904	0.04887
WHI	0.02966	0.00311	0.79987	0.28076

*Summary parameter estimate 0.0289 (SE = 0.0071) *p* = < 0.00001*.

**Q* statistic for heterogeneity with 9 degrees of freedom = 16.66, *p* = 0.0543*.

**Table 6:** **Summary of effect estimates for age by study and estimates of effects in meta-analyses of dropout**.

Study	Parameter estimate	Standard error	Weight in fixed effects model	Weight in random effects model
ACT	−0.01220	0.01223	0.03788	0.11078
LRC	0.01437	0.01063	0.05015	0.11780
DASH	−0.01362	0.02348	0.01028	0.06695
DISC	0.18181	0.25408	0.00009	0.00105
DPP	−0.02086	0.00964	0.06098	0.12205
MRFIT	0.00043	0.00608	0.15329	0.13568
PREMIER	−0.01004	0.01002	0.05644	0.12043
TOHP I	−0.02998	0.01887	0.01591	0.08289
TOHP II	−0.00820	0.01495	0.02535	0.09887
WHI	0.02844	0.00310	0.58964	0.14351

*Summary parameter estimate −0.0033 (SE = 0.0082), *p* = 0.6897*.

*Q statistic for heterogeneity with 9 degrees of freedom = 59.52, *p* < 0.001*.

**Table 7:** **Summary of effect estimates for female gender vs. male by study and estimates of random effects in meta-analyses of dropout**.

Study	Parameter estimate	Standard error	Weight in fixed effects model	Weight in random effects model
ACT	0.52895	0.24405	0.13221	0.14837
LRC	–	–		
DASH	0.18547	0.50574	0.03079	0.05190
DISC	0.41449	0.36164	0.06021	0.08859
DPP	−0.27767	0.18049	0.21473	0.19932
MRFIT	–	–		
PREMIER	0.41463	0.19094	0.21599	0.18994
TOHP I	0.35147	0.26206	0.11467	0.13658
TOHP II	0.18377	0.19628	0.20440	0.18529
WHI	–	–		

*Summary parameter estimate 0.23303 (SE = 0.1251), *p* = 0.0657*.

*Q statistic for heterogeneity with 6 degrees of freedom = 10.77, *p* = 0.0955*.

**Table 8:** **Summary of effect estimates for black race vs. white by study and estimates of random effects in meta-analyses of dropout**.

Study	Parameter estimate	Standard error	Weight in fixed effects model	Weight in random effects model
ACT	0.04334	0.26282	0.03518	0.06413
LRC	−0.34757	0.45305	0.01184	0.02352
DASH	0.18448	0.55379	0.00792	0.01598
DISC	0.16821	0.53303	0.00855	0.01721
DPP	0.39616	0.18966	0.06756	0.11048
MRFIT	0.02200	0.13792	0.12777	0.17541
PREMIER	0.07304	0.17836	0.07640	0.12152
TOHP I	0.02583	0.32479	0.02304	0.04387
TOHP II	−0.31392	0.26873	0.03365	0.06167
WHI	0.29874	0.06302	0.60808	0.36619

*Summary parameter estimate 0.1481 (SE = 0.0711), *p* = 0.0373*.

Q statistic for heterogeneity with 9 degrees of freedom 11.42 *p* = 0.2477

### Pooled analyses

The combined cohort for pooled analyses consisted of 75,764 subjects and the overall dropout percentage was 5.2% (*n* = 3821). The distribution by gender was 53,938 females (71.2%) and 21,826 males (28.8%). The majority were non-Hispanic white (81.9%), followed by non-Hispanic black (11.4%), Asian (3.2%), and Hispanic (1.9%). The mean age was 56.4 years (SD 11.1) and mean baseline BMI was 28.9 (SD = 5.45).

**Figure 1:**
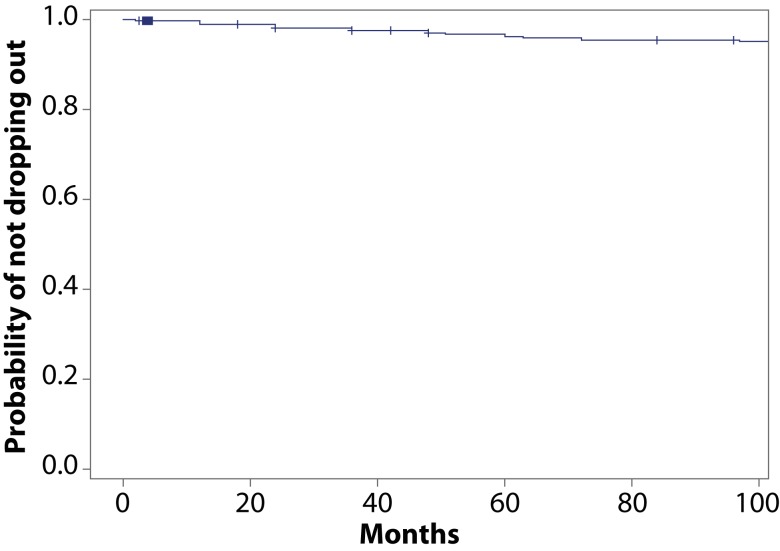
**Study survival probability (by not dropping out of the study) including all subjects from pooled analyses (*N* = 75,764)**.

**Figure 2:**
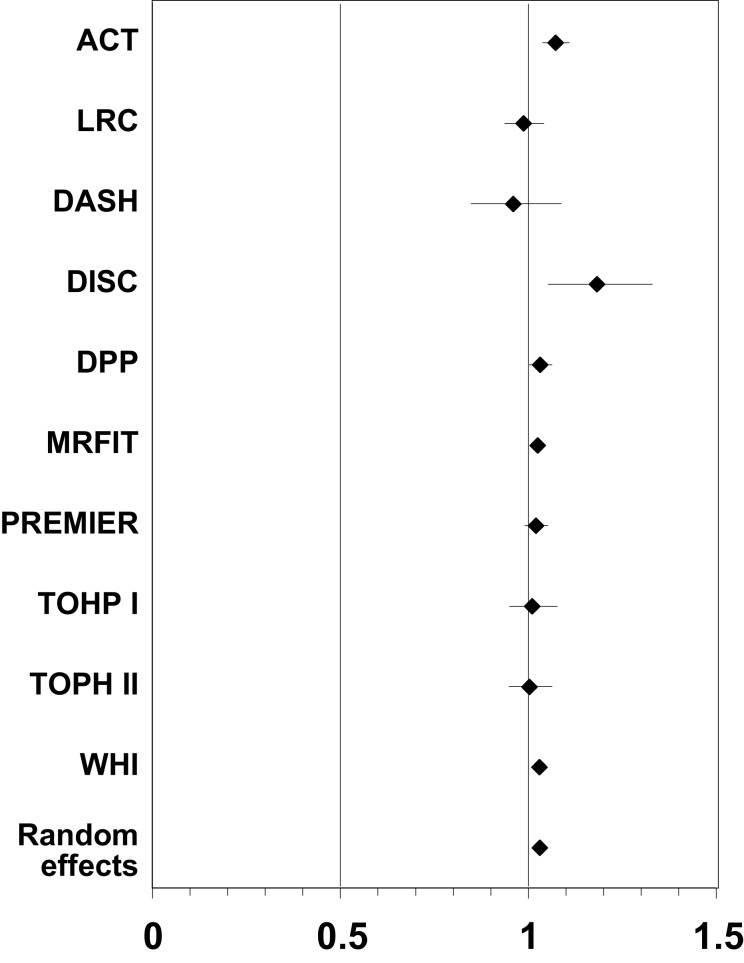
**Hazard ratios of body mass index (BMI) on dropout by study**.

**Figure 3:**
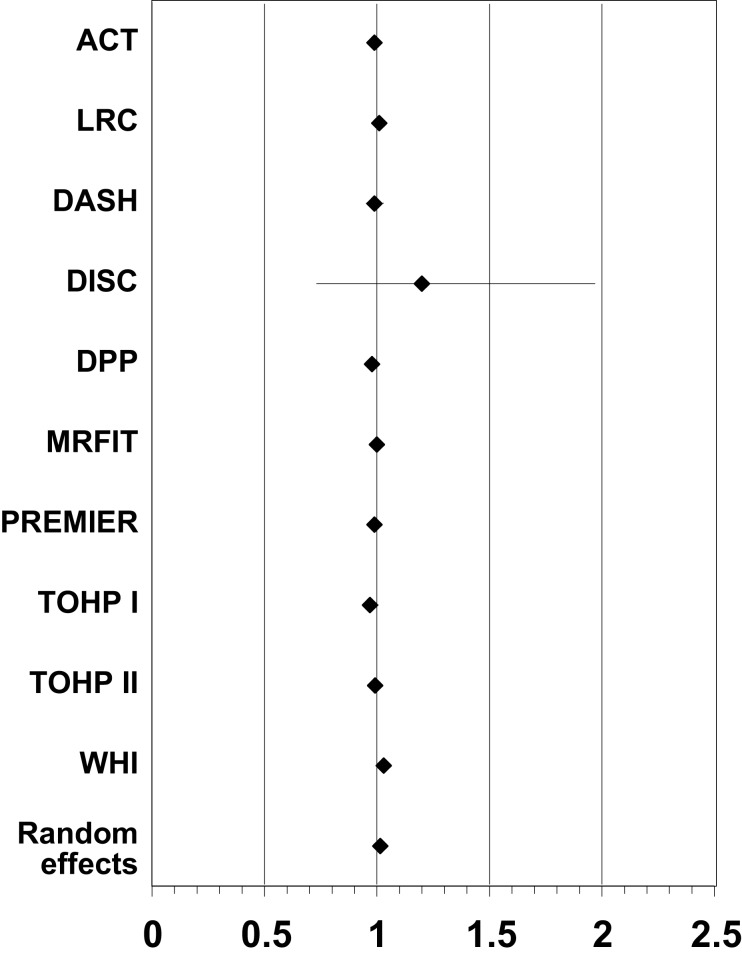
**Hazard ratios of increased age and dropout by study**.

**Figure 4:**
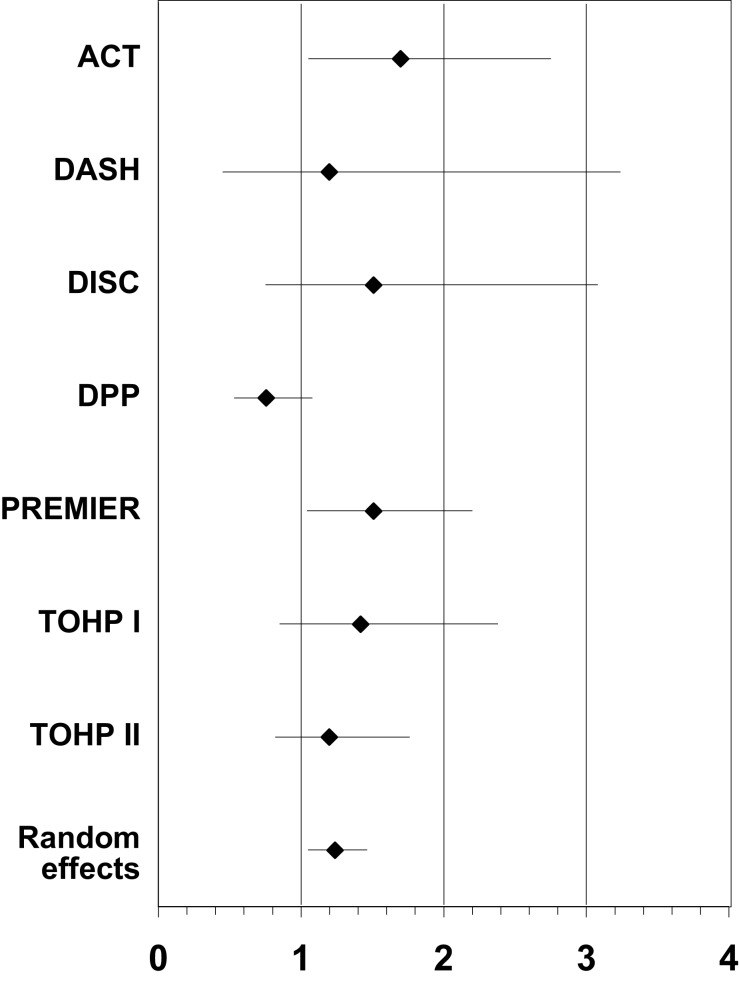
**Hazard ratios of female gender and dropout by study**.

**Figure 5:**
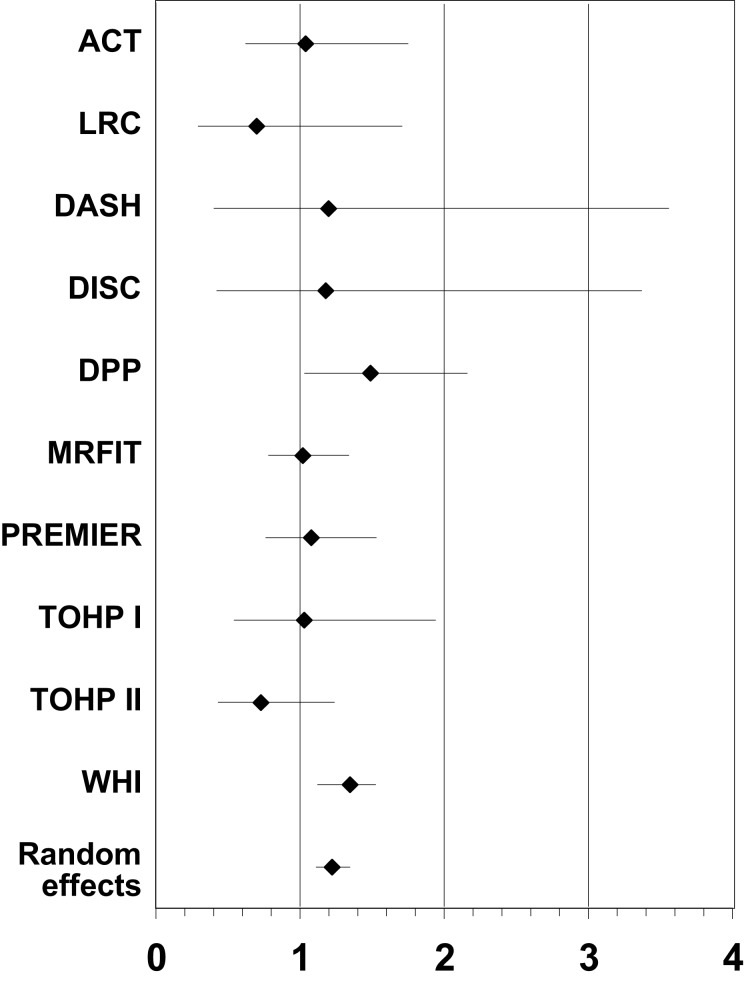
**Hazard ratios of non-white race and dropout by study**.

Results of the multivariate analysis (Table 3) indicate that the risk of dropout was significantly higher for females compared to males (HR = 1.24, 95% CI 1.0, 1.46). Non-Hispanic blacks had a significantly higher dropout rate compared to non-Hispanic whites (HR = 1.22, 95% CI 1.11, 1.35) and Hispanics were also significantly higher compared to non-Hispanic whites (HR = 1.62, 95% CI 1.37, 1.91). There was a statistically significant increased risk of dropout associated with advancing age (Hazard Ratio = 1.01) and increasing BMI (HR = 1.03). The Wald test for the random effect of study was significant (*p* < 0.001). Using age as a categorical variable showed an increased risk of dropout for subjects aged 65 years and over compared to those aged 25–64 years (HR = 1.37; 95% CI = 1.26–1.49). Also individuals who were categorized as obese class II and obese class III (20) were more likely to dropout compared to those who were categorized as overweight or obese class I (HR = 1.40 and 1.69, respectively; Table 4). Figure 1 below shows the combined, overall dropout survival probability using Cox proportional hazards projections. Figures 2–5 show individual study hazard ratios of risk for drop out using BMI, age, gender or race as predictors, respectively.

### Sensitivity analysis

Because there were some inter-study definitions that were not consistent for race [e.g., the DASH study coded race only as minority vs. non-minority and the Lipids Research Clinics (LRC) and PREMIER studies coded race as Black vs. Non-Black], a sensitivity analysis was conducted excluding these studies. An analysis excluding the LRC and PREMIER studies did not show any appreciable difference in the results from the random effects Cox model. A second analysis excluding only the DASH study did not show any significant differences from the combined model (data not shown). Because our analysis included one study of children, who may have differing factors that determine study retention, we performed an additional sensitivity analysis excluding the DISC study. There were no meaningful differences in the results or interpretation following exclusion of the DISC data.

## Discussion

Meta-analysis of individual level data has an advantage; in that, a research question can be addressed that was not part of the original research investigation. By obtaining the individual data, common definitions can be used for coding variables and adjustments for confounders may be performed. Obviously, the power for the meta-analysis is greater than for the individual studies from which the data are compiled. As such, the present analysis may be viewed as presenting very good estimates of the demographic and baseline characteristics that may be associated with increased dropout rates (DORs) in weight loss randomized controlled trials (RCTs).

Some disadvantages of performing a meta-analysis of this type are that the results may not reflect all populations and the meta-analysis itself may be subject to bias because some studies may be excluded due to unavailability of raw data. If internal errors or inconsistency are detected in the analysis, these issues may not be able to be resolved, especially if the data are for public use and tracing back to individual records is not possible. An examination of the survival in study of a small single trial (*N* = 91 at baseline) described the distribution of attrition to be exponential, with an estimated location parameter of Θ = 162 days (meaning 37% remain in the study, 95% CI = 114, 230 days) (21). This example demonstrates a significantly reduced survival on study compared to the large trials that we examined. This difference may be reflective of the differences in resources available between large and small studies. Therefore, our results should be interpreted with caution in terms of applicability to smaller studies. A further limitation of analysis using these types of datasets is the lack of standard ways of coding certain variables often of interest, e.g., marital status (how to analyze “married” vs. “marriage-like relationship”?) and the differing diagnostic criteria used to identify some types of comorbidities. While progress is being made in some arenas of research, more work in standardization remains (22).

Additionally, we focused our analysis on randomized trials of weight loss interventions using traditional diet and/or exercise interventions, which may have very different attrition characteristics than those of observational trials, or of RCTs of weight loss drugs or surgical interventions. Therefore, our results may not be generalizable to these types of analyses due to such reasons as self-selection bias [e.g., as discussed in Ref. (23) who reported higher DORs in younger people] and differing amounts of weight lost that can be observed in drug or surgery trials [e.g., as reported in Ref. (24) who reported younger patients and those with lower BMI at greater risk for dropout of a treatment program]. Weight loss drug trials may have different factors such as side effects, or dissatisfaction with being assigned to the placebo group, causing different retention challenges. Our analysis did not include datasets from these types of trials but hopefully in the future, such data will be made publicly available.

With the presently increasing mean age of the US population, there may be interest in testing weight loss interventions in older samples and researchers should examine and plan for ways to increase study retention in older participants. Further, for studies that will include persons in the obese class II (BMI = 35–39.9) and III (BMI > 40) categories, non-traditional interventions and retention strategies may need to be employed to increase our understanding of the effectiveness of weight loss interventions similar to those we examined. The finding among these datasets that being female was associated with higher DORs cannot be explained by the data provided in the datasets used. Researchers may wish to engage in the practice of performing exit interviews in order to understand the true reasons participants dropped out of the study. Similarly, since there is increasing interest in understanding the racial disparities of obesity in the US, researchers would benefit from designing ways to improve retention and gather regular feedback before a participant drops out of a study so that alternatives can be collaboratively explored.

## Conflict of Interest Statement

The Review Editor Amanda Willig declares that, despite being affiliated to the same institution as authors Kathryn Ann Kaiser, Olivia Affuso, Renee Desmond, and David B Allison, the review process was handled objectively and no conflict of interest exists. The authors declare that the research was conducted in the absence of any commercial or financial relationships that could be construed as a potential conflict of interest.
